# 金属有机骨架衍生材料在样品前处理中的应用研究进展

**DOI:** 10.3724/SP.J.1123.2021.05017

**Published:** 2021-09-08

**Authors:** Wenmin ZHANG, Qingqing LI, Min FANG, Jia GAO, Zongbao CHEN, Lan ZHANG

**Affiliations:** 1.闽江师范高等专科学校, 福建 福州 350108; 1. Minjiang Teachers College, Fuzhou 350108, China; 2.福州大学, 食品安全与生物分析教育部重点实验室, 福建 福州 350116; 2. Ministry of Education Key Laboratory for Analytical Science of Food Safety and Biology, Fuzhou University, Fuzhou 350116, China

**Keywords:** 金属有机骨架, 衍生化材料, 前处理技术, 综述, metal-organic frameworks, derivative materials, pretreatment technique, review

## Abstract

样品前处理技术在复杂样品的整个分析过程中起着至关重要的作用,其不仅可以提高痕量目标物在样品中的浓度,而且能有效消除样品基质对分析的干扰。对于样品前处理技术而言,吸附剂是其最为核心部分。因此开发高效、稳定的新型吸附剂已成为前处理技术领域的研究热点。近年来,由金属有机骨架(metal-organic frameworks, MOFs)衍生的多孔材料因其形貌结构多样、孔径可调、比表面积高、热稳定性良好、耐化学腐蚀等优异性能,使其在样品前处理领域拥有广阔的应用前景,基于MOFs衍生材料的样品前处理新方法也层出不穷。然而,MOFs衍生材料仍存在MOFs前驱体合成工艺复杂、生产成本高、量产困难等问题。该文总结了近几年来MOFs衍生材料在分散固相萃取(dSPE)、磁固相萃取(MSPE)、固相微萃取(SPME)、搅拌棒固相萃取(SBSE)和分散微固相萃取(DMSPE)等样品前处理技术中的研究进展,并对多种MOFs衍生材料的制备方法、功能化调控、富集效率等方面进行了评述。最后,展望了MOFs衍生材料在该领域中的应用前景,为进一步研究MOFs衍生材料的应用提供了参考。

## Abstract (en)

Sample pretreatment technology plays a vital role throughout the analysis of complex samples. Sample pretreatment can not only increase the concentration of trace targets in the sample, but also effectively eliminate interference from the sample matrix in instrumental analysis. Adsorbent materials are a key component of sample pretreatment technology. Therefore, the development of efficient and stable new adsorbent materials has acquired significance in research on pretreatment technology. Porous materials are advantageous for use in diverse applications, such as in adsorbents, when they possess controllable nanostructures, a tailored pore surface chemistry, and abundant porosity, and are inexpensive. Particularly in recent years, porous materials derived from metal-organic frameworks (MOFs) feature excellent properties, such as diverse morphology and structure, adjustable pore size, high specific surface area, good thermal stability, and chemical resistance. MOF-derived materials, when used as adsorbents for sample pretreatment, offer the following advantages: (1) The porous materials derived from MOFs typically possess a larger specific surface area than other porous materials. This characteristic is beneficial to improve the extraction capacity and extraction efficiency via an increase in the contact area between the materials and targets; (2) The microscopic porous structure of MOF-derived materials can be easily tuned (by controlling the temperature and time during pyrolysis, gas atmosphere, and heating rate), which is conducive to improve the selectivity of sample pretreatment methods; (3) The metal active sites can be evenly distributed. Owing to the ordered distribution of metal ions in the precursor MOFs and a good periodic framework structure, the metal active sites of the derivatives formed can still maintain a corresponding distance. These metal active sites will not form agglomerates and affect the extraction performance; conversely, other porous materials often require extremely complicated processes to achieve a uniform distribution; (4) Heteroatoms such as nitrogen and sulfur can be easily doped on the framework of MOF-derived porous materials. This doping enables the materials to induce additional interactions such as hydrogen bonding and *π-π* stacking for adsorbing target analytes. The excellent properties of MOF-derived materials make them promising for use in sample pretreatment. Novel sample pretreatment methods that use MOF-derived materials are constantly being developed. However, the use of MOF-derived materials is limited by the complex preparation process and high production cost of MOF precursors, along with difficulties in mass production. Further, the precise design or functionalization of MOF-derived materials according to the characteristics of targets is a new direction with immense challenges as well as application potential. This review summarizes the application of MOF-derived materials in sample pretreatment methods, including dispersive solid phase extraction (dSPE), magnetic solid phase extraction (MSPE), solid phase microextraction (SPME), stir bar sorptive extraction (SBSE), and dispersive micro solid phase extraction (DMSPE). The preparation methods, functional control, and enrichment efficiencies of various MOF-derived materials are also reviewed. Finally, the application prospects of MOF-derived materials in sample pretreatment are discussed to provide a clear outlook and reference for further related research.

前处理技术在复杂样品(如生物、食品和环境等样品)的整个分析过程中起着至关重要的作用。对复杂样品进行适当的前处理,不仅可以将痕量目标分析物富集以达到可检测的最低限度,而且能有效消除样品基质对仪器分析的干扰。各种前处理技术如固相萃取、固相微萃取、磁固相萃取、搅拌棒固相萃取和管内固相萃取等,在复杂样品的前处理中发挥了重要的作用^[1]^。在这些前处理技术中,吸附剂的选择是其中的关键因素。开发高效、稳定的新型吸附剂已成为前处理技术领域的研究热点。

金属有机骨架(metal-organic frameworks, MOFs)是一种由无机金属离子(或金属簇)作为节点或者中心,与有机配体通过自组装的方式形成的具有周期性和无限延伸骨架结构的多孔晶体材料^[2,3]^。其具有高比表面积、高孔隙率、较好的化学和物理稳定性以及其结构的可调性,已经广泛应用在储能^[4]^、催化^[5]^、药物^[6]^和分离^[7]^等领域。

由于MOFs材料中金属离子与有机配体间的配位键在外部环境的影响下不稳定,因此在高温以及不同气氛条件下,MOFs可以作为多孔碳材料或金属氧化物/碳复合材料的合成前驱体。2008年,Xu等^[8]^首次以MOF-5为前驱体,通过直接一步热解法成功制备了具有多孔特性的碳材料,该研究成果得到了广泛关注。作为一种具有牺牲模板和金属前体的双功能材料,MOFs在构建具有独特多孔结构和功能性外壳的纳米多孔碳材料中发挥重要作用^[9]^。由MOFs衍生的多孔碳材料比其他碳基材料具有更高比表面积,并且可以保留其前驱体MOFs的原始形貌^[10]^、孔隙率和化学成分,或者会演变为更有趣的结构,如碳纳米片^[11]^、碳纳米管^[12,13]^以及碳纳米棒^[14]^。

MOFs衍生物作为一种制备简单的多孔碳材料,用作样品前处理的吸附剂,有以下的优势:(1)由MOFs衍生的多孔碳材料一般拥有比其他碳基材料更大的比表面积,有利于提高材料与目标物的接触面积,提高萃取容量和萃取效率;(2)其微观多孔结构易调(控制热解过程中的温度和时间、气体氛围和升温速率等),有利于提高吸附的选择性;(3)金属活性位点可以实现均匀分布。由于前驱体MOFs中金属离子的有序分布以及良好的周期性骨架结构,其所形成衍生物的金属活性位点仍可以保持相应的距离,不会形成团聚物从而影响萃取性能,而其他多孔碳材料要实现这种均匀的分布,往往需要极为复杂的操作^[15]^; (4)氮和硫等杂原子容易掺杂在MOFs衍生的多孔碳骨架上^[16]^,这些杂原子的掺杂使得MOFs衍生物在吸附目标分析物时会产生额外的氢键作用和*π-π*堆积作用。这些优异的性能,使MOFs衍生物十分适合用作吸附剂,在样品前处理领域有着巨大的发展潜力。

本文将结合近年来本课题组以及国内外其他研究者报道的相关研究工作,对MOFs衍生物的制备以及基于MOFs衍生物的前处理技术进行总结和评述,并对其发展前景进行了展望。

## 1 MOFs衍生物在分散固相萃取领域的应用

分散固相萃取(dispersive solid phase extraction, dSPE)是基于柱固相萃取技术发展起来的一种前处理方法,其将固相萃取吸附剂直接分散于样品的粗提取液中,通过吸附剂与粗提取液的充分接触,高效富集溶液内的目标分析物,然后利用离心或过滤等方式将吸附剂与溶液分离,最后使用合适的溶剂将固相吸附剂上的目标分析物洗脱下来。例如,Liu等^[17]^将ZIF-8在氮气氛围下热解碳化,得到了ZIF-8衍生的多孔碳材料(MOF-C)。MOF-C保留了前驱体ZIF-8的微孔结构,比表面积可达1320 m^2^/g。随后将该材料用作dSPE的吸附剂,并结合高效液相色谱-紫外检测器(HPLC-UV),用于检测环境水和水果样品中的苯甲酰脲类杀虫剂,检出限为0.1~0.23 ng/mL。Wang等^[18]^制备了一种对氟喹诺酮类药物有高富集能力的铜-石墨碳笼材料。该材料由Cu-MOFs中的HKUST-1在氮气氛围下热解得到。该材料中Cu纳米颗粒可均匀地分散在八面体的碳基底内部,并且其表面具有超薄的石墨层,使得铜-石墨碳笼材料与目标分析物有*π-π*堆积相互作用。另外,铜-石墨碳笼材料中的含氧基团与氟喹诺酮类药物中的羟基有强烈的氢键作用。将该材料作为dSPE的吸附剂,结合超高效液相色谱-紫外检测法(UPLC-UV)建立了环境水样品和食品样品中氟喹诺酮类药物的分析方法,检出限和线性范围分别为0.018~0.042 ng/mL和0.1~500 ng/mL。

吸附剂在液体样品中的良好分散性可以增大其与目标分析物的有效接触面积,从而提高吸附效率。包括MOFs衍生物在内的多数多孔碳材料在水溶剂中的分散性都较差,极易形成团聚或缠绕^[19]^,这在一定程度上限制了MOFs衍生物在样品前处理中的应用。

在多孔碳材料表面修饰一些亲水性基团是一个解决其分散性问题的有效方案。由于MOFs自身往往会带有一些亲水性官能团(羧基和羟基等),因此在碳化过程中,这些官能团可以有效保留在多孔碳的表面,从而增强材料的水中分散性^[20,21]^。例如,Taghvimi等^[22]^将ZIF-8碳化,制备了一种对甲基苯丙胺有强富集能力的羧基化多孔碳材料。该材料表面带有负电荷的羧基不仅增强了其在液体中分散性,还能与甲基苯丙胺中带正电荷的氨基产生强烈的静电作用,大大提高了吸附效率。该作者将羧化多孔碳材料用作分散固相萃取的吸附剂,结合HPLC-UV技术建立了尿液中甲基苯丙胺的分析方法,检出限和线性范围分别为10 ng/mL和50~2500 ng/mL。

此外,氮原子的掺杂也可以有效增加材料的亲水性。制备富含氮的MOFs衍生物主要有两种方法,一种是由含氮的MOFs(其氮原子通常由有机配体提供)直接碳化得到,另一种方法是在MOFs中引入外部氮源,例如将MOFs浸泡在尿素、三聚氰胺、离子液体和一水合氨等溶液中,然后再进行碳化得到氮掺杂的MOFs衍生多孔碳材料^[23]^。例如,Liu等^[24]^制备了一种由ZIF-8衍生的含氮多孔碳(ZIF-8-NC)材料,并将其作为金属离子吸附剂。该材料表面存在的含氮基团不仅改善了材料的分散性,并且可以与金属离子产生配位作用,因此对金属离子有很好的吸附性能。将其与火焰原子吸收光谱法(FAAS)相结合,成功用于茶样品中痕量Cu^2+^、Pb^2+^、Cd^2+^、Cr^3+^、Ni^2+^、Co^2+^的分析,该方法的检出限为0.05~0.07 μg/kg。Ghorbani等^[25]^将ZIF-67沉积在ZIF-8的表面,制备了一种核-壳结构的双ZIF材料,并将其热解,得到这种双ZIF材料衍生的含有Zn和Co金属颗粒的氮掺杂多孔碳(Zn/Co/C)材料。该材料对Pb(Ⅱ)、Cr(Ⅲ)和Cr(Ⅵ)的吸附容量可分别达到490、520和500 mg/g。

## 2 MOFs衍生物在磁固相萃取领域的应用

磁固相萃取(magnetic solid phase extraction, MSPE)技术是1996年由Towler等^[26]^首次提出的操作简便、环境友好、传质速率快、吸附剂材料易回收的一种前处理方法^[27]^。该方法直接将磁性吸附剂加入到样品溶液中以吸附目标分析物,待吸附完成后,通过施加外部磁场直接将磁性吸附剂与样品溶液分离,不需要过滤或离心步骤,使得吸附剂与样品的分离更加快速简单,有效避免了传统柱固相萃取中吸附剂与样品分离的耗时操作。最后将萃取了目标分析物的磁性吸附剂分散在适当的洗脱溶液中,同样通过磁性将其与洗脱液进行分离,收集洗脱液进样分析。MSPE的核心是磁性吸附剂,制备磁性材料的方法主要是将具有磁性的纳米颗粒(如四氧化三铁)利用物理混合、包埋和层层生长等方法修饰到材料上。然而这些磁性材料的制备方法往往都需要复杂的步骤,导致磁性材料批次间的重复性较差。另外,许多磁性材料经过层层的修饰之后,其磁性大大减弱,直接影响了吸附剂和溶液的分离效率。因此,研究一种制备简单、易于调控的新型磁性材料能有效促进MSPE技术的发展。

### 2.1 磁性金属中心MOFs衍生物的研究与应用

利用一些自身就具备磁性金属中心的MOFs通过简单的一步热解法即可得到磁性多孔碳材料,这些磁性金属离子在高温热解过程中可以以纳米颗粒的形式保留在多孔碳中,有效避免了传统方法制备磁性材料的步骤复杂和磁性降低等问题。2014年,Lin等^[28]^首次将ZIF-67(由钴阳离子和2-甲基咪唑阴离子连接而成)衍生的磁性Co基纳米多孔碳材料用作MSPE的吸附剂,实现了环境水和甜瓜样品中新烟碱类杀虫剂的富集,从此MOFs衍生物的应用便成了MSPE领域的研究热点之一。

例如,Liu等^[29]^在氮气氛围中碳化ZIF-67,制备了一种磁性多孔碳纳米材料,Co纳米颗粒的存在使制备的碳纳米材料具有良好的孔隙率、较高的比表面积以及优异的磁性能。将该材料作为MSPE的吸附剂,并结合HPLC-UV建立了葡萄和苦瓜样品中苯脲类除草剂的分析方法,检出限和线性范围分别为0.17~0.46 ng/g和1.0~100.0 ng/g。Duo等^[30]^制备了一种磁性层状镍/氧化镍/碳纳米棒(Ni/NiO@C)材料,该材料由Ni-MOFs在氩气氛围下热解制得,保持了原始Ni-MOFs的棒状形貌,具有磁性的Ni/NiO纳米颗粒均匀地分散在中孔结构的碳材料中。将该材料作为MSPE的吸附剂,结合HPLC-UV技术,实现了食品中苯甲酰脲类杀虫剂的分离分析。Duo等^[31]^在氩气氛围下碳化MOF-235(属于Fe-MOFs)制备了一种Fe_2_O_3_@C材料,该材料中Fe_2_O_3_均匀地嵌入衍生的多孔碳基质中,具有良好的磁性。将Fe_2_O_3_@C作为MSPE的吸附剂对苯甲酰脲类杀虫剂进行富集,其强的*π-π*相互作用以及疏水相互作用增强了对苯甲酰脲类杀虫剂的亲和性,结合HPLC-UV技术建立了茶样品中苯甲酰脲类杀虫剂的分析方法,该方法预处理时间短(20 min),检出限低(0.05~0.1 μg/mL),线性范围为0.2~450 μg/mL。

上述这些磁性碳材料中,磁性金属纳米颗粒往往只是散布在多孔碳基质中,在一些极端条件下,磁性金属纳米颗粒可能会受到损害从而降低了材料的磁性。为了获得磁性更加稳定的多孔碳材料,对磁性金属纳米颗粒进行适当的保护是有必要的。

Zhang等^[32]^利用ZIF-67在氩气/氢气混合氛围下制备了一种保留有前驱体十二面体形态的氮掺杂碳纳米管笼(N-CNTCs),其粒子表面由相互连接的碳纳米管组成。值得注意的是,在热解过程中,ZIF-67中的Co阳离子被还原成磁性Co纳米颗粒,被碳纳米管捕获并封装在其内部。并对N-CNTCs的磁稳定性进行了考察,结果表明酸浸后N-CNTCs的磁响应仅下降10%,而碱浸后仅下降5%,在这种极端条件下仍能保持良好磁性的原因主要是纳米管保护了封装在其中的磁性Co纳米粒子,使其免受酸或碱的腐蚀。因此即使在极端的条件下,制备的N-CNTCs也可以在外部磁场下实现快速的固液分离。另外,该材料中氮掺杂含量高达15.42%,远高于大多数多孔碳材料,高含量的氮掺杂不仅增加了材料的分散性,还提供了如氢键作用和*π-π*堆积作用等其他分子间作用力。相比于没有氮掺杂的碳材料,磁性N-CNTCs材料对冈田海绵酸的吸附性能提高了50%。以N-CNTCs为MSPE的萃取材料,结合HPLC-MS/MS建立了水产品中冈田海绵酸的分析方法。该法的检出限低(1.3 pg/mL),重现性好,回收率在82.0%~107.0%之间,且不受常见的盐离子和海鲜样品中其他基质的干扰。Wu等^[33]^通过在氩气氛围下煅烧ZIF-67,将其转化为一种含Co纳米颗粒的磁性碳纳米管(Co@CNTs)。该材料中Co同样被封装在碳纳米管的内部。将Co@CNTs作为MSPE的吸附剂,在*π-π*相互作用和范德华力的相互作用下,Co@CNTs对氟比洛芬和酮洛芬具有优越的萃取效率,其吸附能力分别为1176.5 mg/g和1226.5 mg/g;结合HPLC-UV技术建立了人体血清样品中氟比洛芬和酮洛芬的分析方法,该法前处理时间短(10 min),检出限低(0.6~0.7 ng/L),回收率在86.74%至97.22%之间,且分析不受血清样品基质的干扰。

### 2.2 非磁性金属中心MOFs衍生物的研究与应用

目前,只有少数的MOFs系列包含有磁性的金属中心,探究非金属中心MOFs衍生物的磁性功能化,将有助于扩大MOFs衍生物在MSPE领域的应用。例如,由Zn-MOFs衍生的多孔碳材料一般都具有较大的比表面积^[34]^(最大可达3453 m^2^/g),远高于其他磁性金属中心MOFs衍生物(如Co-MOFs的衍生物比表面积通常只有200~300 m^2^/g),但是由于属于非磁性金属中心,单一的Zn-MOFs衍生物往往缺乏磁性。因此,怎么赋予Zn-MOFs衍生物磁性,以利用其较大的比表面积引起了研究人员的兴趣。Liu等^[35]^制备了一种由ZIF-8和ZIF-67组合形成的双金属ZIF(BMZIF)材料,该材料结合了ZIF-8和ZIF-67的结构特性,可作为制备稳定性好、磁性强和孔隙率高的多孔碳材料前驱体。将双金属BMZIF碳化,得到BMZIF衍生的磁性多孔碳材料,该材料比表面积为396 m^2^/g,将其作为MSPE的吸附剂,结合气相色谱-质谱(GC/MS)技术建立了水样品中有机氯农药(OCP)的分析方法,可应用于自来水、废水、茶水以及梨汁中有机氯农药的测定,检出限和线性范围分别为0.39~0.70 ng/L和2~500 ng/L。

为了获得比表面积更大的磁性Zn-MOFs衍生物,Wang等^[36]^将Co掺杂进ZIF-8的骨架中,制备了一种Co/ZIF-8双金属MOFs材料,通过进一步将Co/ZIF-8碳化,得到Co掺杂的磁性分层多孔碳(Co/HPC)材料,并且探讨了MOFs前驱体中Co掺杂含量对衍生物磁性以及比表面积的影响。实验结果表明,Co的含量过少时,虽然得到的Co/HPC比表面积较大,但是其磁性较弱,不适用于作为MSPE的吸附剂;同样,当Co含量较高时,Co/HPC的磁性较强,但是其比表面积则较小。鉴于此,该作者最终选择了Co/Zn物质的量比为7:1的Co/ZIF-8作为前驱体制备Co/HPC。在该比例下得到的Co/HPC材料拥有大的比表面积(715 m^2^/g)、较强的磁性以及良好的稳定性。将Co/HPC作为MSPE的吸附剂,结合HPLC-DAD技术,建立了水样和白葫芦样品中三嗪除草剂的分析方法,检出限和线性范围分别为0.02 ng/mL和0.2~100 ng/mL。

制备非金属磁性中心MOFs衍生的磁性多孔碳材料,除了上述调控磁性金属颗粒的掺杂量外,还可以通过先制备非金属磁性MOFs衍生物,随后利用磁性金属盐将其磁化。例如,Liu等^[37]^在氩气氛围下热解MOF-5(Zn-MOFs),得到MOF-5-C,随后使用铁盐与MOF-5-C进行共沉淀以引入磁性,最终得到一种由MOF-5衍生的磁性多孔碳(MPC)材料。该材料具有良好的多孔结构和磁性,比表面积可达1058 m^2^/g。将其用作MSPE的吸附剂,结合HPLC-UV,建立了苹果样品中4种氨基甲酸酯的分析方法,检出限和线性范围分别为0.1~0.2 ng/g和0.5~100 ng/g。

## 3 MOFs衍生物在固相微萃取领域的应用

固相微萃取(solid phase microextraction, SPME)是Pawliszyn课题组^[38]^在1989年首次提出的一种样品前处理技术,该技术将石英丝或者金属丝制成SPME纤维,随后在纤维的一端涂覆上吸附剂,利用分析物在该吸附剂上的吸附和解吸原理进行萃取^[39,40,41]^。与传统的样品前处理技术相比,SPME所需的吸附剂量少,可以集取样、萃取、浓缩于一体。在SPME中,涂层材料的选择是极其重要的。目前对SPME的研究主要是集中于开发新型的吸附剂涂层材料上,希望涂层材料在提供高萃取效率、高选择性的同时,还能保持良好的机械和化学稳定性^[42]^。Zhang等^[43]^率先将MOFs衍生物应用于SPME的研究。他们以Al-MOFs为前驱体,在氮气氛围下热解得到C-Al-MOF材料,并通过溶胶-凝胶法制备了C-Al-MOF涂层纤维,结合GC-MS,对水和土壤样品中的多环芳烃(PAHs)进行了高灵敏检测。Zhang等^[44]^以ZIF-67为前驱体制备了钴纳米多孔碳(Co-NPC),并以物理黏合的方法将其涂覆在不锈钢丝上,实现了蔬菜样品中5种有机氯农药(OCPs)的高效富集;基于*π-π*堆积相互作用和疏水相互作用,结合气相色谱-微电子捕获检测器(GC-μECD)建立了OCPs的分析方法,检出限为0.07~0.45 ng/g,线性范围为0.3~50 ng/g。

萃取时间的缩短对于整个SPME过程效率的提高有重要意义。由于大的比表面积以及较快的传质效率,具有中空结构的纳米材料在提高吸附平衡和吸附能力方面起着重要作用,是一种有潜力的吸附剂。以MOFs为模板制备中空碳材料操作简单,合成快速。Hu等^[45]^分别以ZIF-8和空心ZIF-8为前驱体,制备了碳纳米立方体(CNC)和空心碳纳米立方体(HCNC)。制备的两种碳材料均通过物理黏合法涂覆在不锈钢丝表面,随后对两种纤维富集PAHs的性能进行了对比。由空心结构引起的丰富活性位点以及疏水相互作用,HCNC涂层纤维表现出更优异的富集性能。Zhang等^[46]^以ZIF-67为前驱体制备了一种具有“空心笼”结构的氮掺杂碳纳米笼(N-CNTCs)材料,并以物理黏合的方式涂覆在不锈钢丝表面,将所得到的N-CNTCs涂层纤维与GC-MS技术相结合,建立了环境水样中多氯联苯(PCBs)的分析方法,检出限为0.10~0.22 ng/L,线性范围为0.3~1000.0 ng/L。由于空心笼状结构的传质长度减少,N-CNTCs涂层纤维的传质速率更快,与实心结构的氮掺杂碳(SNC)纤维相比,N-CNTCs包裹的纤维提取多氯联苯的平衡时间缩短了10~20 min。另外,大量的氮掺杂也为吸附提供了额外的氢键作用,大大提高了萃取效率。

材料的形貌对萃取性能也有很大的影响。凭借MOFs形貌易于调控的优点,可以制备相应形貌的MOFs衍生碳材料,以满足不同的应用需求。例如,Hu等^[47]^制备了一种Co&thiourea@MIL-101-NH_2_衍生的海胆状纳米孔碳(Co&thiourea@MIL-101-NH_2_-derived NPC),并采用物理黏合的方法将其固定在不锈钢丝上,结合GC-MS建立了环境水样中苯系物(BTEX)的分析方法,检出限为0.08~0.36 ng/L。Co&thiourea@MIL-101-NH_2_-derived NPC中独特的海胆形貌提供了更大的比表面积、更多的有效吸附位点以及有利于传质的孔结构。

### 3.1 MOFs衍生的金属氧化物/碳复合材料

由于良好的热稳定性、机械稳定性、化学稳定性以及可能的分子筛效应和其他化学机理作用^[48]^(如M-S键),金属氧化物如ZnO、TiO_2_、PbO_2_、Co_3_O_4_和Al_2_O_3_等^[49,50,51,52,53]^在SPME领域已经有了广泛的研究。但是金属氧化物由于易于团聚^[54]^以及比表面积较小等问题,其在SPME中的应用受到了一定的限制。将金属氧化物进一步负载在碳基底上,不仅可以防止其团聚,还可以增大材料的比表面积,是一个有效的解决方法。在MOFs的热解过程中可以通过控制温度和气体氛围,将MOFs中的金属离子转化为金属氧化物并且负载在衍生的多孔碳基底上,这种金属氧化物/碳复合材料的制备方法简便易行,有望扩展金属氧化物在SPME领域中的应用。

Saraji等^[55]^以Zr-MOFs为前驱体,在N_2_氛围下热解制备了介孔碳-ZrO_2_复合材料,通过物理黏合法将该材料涂覆在不锈钢丝表面,结合气相色谱-火焰离子检测器(GC-FID),实现了水样中BTEX的分析与检测,方法的检出限为50~560 ng/L。目前,从环境污染物中高效萃取具有不同性质的目标物仍然是一个很大的挑战,因此,开发一种可应用于富集广谱目标分析物的吸附剂引起了研究人员极大的兴趣^[56,57]^。Hu等^[58]^制备了一种ZIF-8衍生的双壳空心ZnO/C材料,并通过物理黏合法制备了ZnO/C涂层的不锈钢丝,该纤维可用于富集极性以及非极性的目标分析物。通过与GC-MS结合,建立了水样中苯系物(BETX)和氯酚(CPs)的分析方法,检出限为0.14~0.56 ng/L(BETX)和1.10~2.84 ng/L(CPs)。ZnO/C材料中的Zn-OHs为吸附CPs提供了额外的氢键相互作用,而碳材料则通过*π-π*相互作用和疏水相互作用吸附BTEX,两者之间的协同相互作用赋予了其有效萃取广谱污染物的能力。另外,双壳的空心结构也为ZnO/C涂层纤维萃取目标分析物提供了较快的传质速率,改善了萃取性能,其萃取能力是商业PDMS/DVB纤维的7~32倍。

### 3.2 原位碳化法制备基于MOFs衍生物的SPME纤维

在SPME技术中,除了对涂层材料的选择之外,对涂层方法的探究也尤为重要,涂层方法会影响纤维的使用寿命和重现性等。目前制备MOFs衍生物涂层纤维的方法大多还是使用传统的物理黏合法,需要额外的涂层步骤,但是由于MOFs自身可以通过原位水热法生长在纤维表面,而MOFs又可以通过一步碳化法转化为多孔碳材料,因此如何利用二者之间的关系以简便地制备MOFs衍生物涂层纤维引起了研究人员的兴趣。

Wei等^[59]^开发了一种简便的原位碳化法用以制作MOFs衍生物涂层纤维。该法通过水热反应在不锈钢纤维上原位生长MOF-74涂层,再将其放置于管式炉中以实现MOF-74的碳化,最终直接得到MOF-74-C涂层纤维。该纤维被用于富集环境水样中的芳香型污染物,纤维的使用寿命达130次以上。由于MOF-74-C涂层的微孔结构(0.59至1.71 nm)产生了尺寸排阻效应,因此其拥有很高的选择性,可以排除环境水样品中其他溶解有机物的影响。由于原位水热生长法制备的涂层材料与纤维基底之间缺少强有力的连接,因此在原位碳化制备MOFs衍生物涂层纤维之前,强化MOFs与纤维基底的连接是十分有必要的。Du等^[60]^首先用镍/钛合金的基底纤维进行水热处理,以获得镍/钛氧化物纳米薄片,接着用电沉积的方法制备了Co涂层,并将Co涂层用于原位水热生长ZIF-67,最后将纤维放置于管式炉中碳化后,制得Co@ZIF-67-C涂层纤维。由于沉积的Co涂层具有丰富的成核位点,可用于后续2-甲基咪唑与Co^2+^离子之间的配位反应,增强了ZIF-67与镍/钛合金纤维间的连接,从而有效延长了原位碳化法制备纤维的使用寿命。将制备的Co@ZIF-67-C涂层纤维与HPLC-UV技术相结合,建立了环境水样中多环芳烃的分析方法,检出限为5~45 ng/L,使用寿命达150次以上。

## 4 MOFs衍生物在其他前处理技术中的应用

除了固相萃取、磁固相萃取和固相微萃取之外,MOFs近两年来也开始应用到其他前处理技术中。搅拌棒固相萃取(stir bar sorptive extraction, SBSE)的萃取原理与SPME基本相同,都是基于目标分析物在固定相和水相之间吸附与解吸的分配平衡,但是SBSE中涂层材料的涂覆量是SPME的50~250倍^[61]^,因此具有更高的萃取效率。另外,SBSE中不需要在待测样品中添加搅拌磁子,从而避免了磁子对目标分析物的竞争性吸附。Ghani等^[62]^以ZIF-67为前体制备了一种Co纳米多孔碳(Co-NPC)材料,并通过物理胶粘的方法制备了Co-NPC的SBSE涂层搅拌棒,涂层厚度约为125 μm。将制得的搅拌棒与HPLC-UV联用,实现了人体尿和血样品中氟尿嘧啶和苯巴比妥的测定,检出限和线性范围分别为0.21~0.36 μg/L和1~500 μg/L。

分散微固相萃取(dispersive micro solid phase extraction, DMSPE)是Lehotay课题组^[63]^提出的一种小型化的固相萃取技术,其分散在样品溶液中的固体吸附剂含量在微克范围内^[64]^,具有简单、快速、溶剂消耗低的优点。Gonzalez等^[65]^以MOF-67衍生的磁性多孔碳(MPC)为吸附剂,发展了一种自动化DMSPE技术。MPC高的孔隙率、良好的稳定性以及分散性促进了DMSPE的自动化,在分析具有复杂基质的样品时,不会出现背压或仪器导管堵塞等问题。他们结合GC-MS技术建立了环境水样品中雌激素的分析方法,该法预处理时间短(低于20 min), DMSPE自动化系统拥有良好的精度,相对标准偏差(RSD)为2.7%~5.9%,回收率为86%~115%。

表1总结了近几年来MOFs衍生物在样品前处理中的制备与应用。

**表1:** MOFs衍生物在样品前处理中的应用

Material	Precursor		Analyte		Method		Technique		Sample		Reference
MOF-C	ZIF-8		benzoylurea		dSPE		HPLC-UV		water and fruits		[17]
Cu@graphitic octahedron carbon cages	Cu_3_(BTC)_2_		fluoroquinolones		dSPE		UPLC-UV		water and food		[18]
Carboxylated carbon porous	ZIF-8		methamphetamine		dSPE		HPLC-UV		biological urine		[22]
ZIF-8-NC	ZIF-8		metal ions		dSPE		FAAS		tea		[24]
Zn/Co/C	ZIF-8/ZIF-67		metal ions		dSPE		FAAS		water		[25]
MNC	ZIF-67		phenylurea herbicides		MSPE		HPLC-UV		food		[29]
Ni/NiO@C	Ni-MOF		benzoylurea insecticides		MSPE		HPLC-UV		food		[30]
Fe_2_O_3_@C	MOF-235		benzoylurea insecticides		MSPE		HPLC-UV		tea		[31]
N-CNTCs	MOF-67		okadaic acid		MSPE		HPLC-MS/MS		seafood		[32]
Co@CNTs	ZIF-67		flurbiprofen and ketoprofen		MSPE		HPLC-UV		human serum		[33]
BMZIF-derived magnetic porous carbon	BMZIF		organochlorine pesticides		MSPE		GC/MS		water		[35]
Material		Precursor		Analyte		Method		Technique		Sample	Reference
Co/HPC		Co/ZIF-8		triazine herbicides		MSPE		HPLC-DAD		water and plants	[36]
MOF-5-C		MOF-5		carbamate		MSPE		HPLC-UV		fruits	[37]
C-Al-MOF		Al-MOF		PAHs		SPME		GC-MS		water and soil	[43]
Co-NPC		ZIF-67		organochlorine pesticides		SPME		GC/μECD		vegetables	[44]
HCNC		hollow-ZIF-8		PAHs		SPME		GC/MS		water	[45]
N-CNTCs		ZIF-67		PCBs		SPME		GC/MS		water	[46]
Co&thiourea@MIL-101-NH_2_- derived NPC		Co&thiourea@ MIL-101-NH_2_		BTEX		SPME		GC-MS		water	[47]
Mesoporous carbon-ZrO_2_		Zr-MOF		BTEX		SPME		GC-FID		water	[55]
Double-shelled hollow ZnO/C		ZIF-8		BTEX and chlorophenols		SPME		GC-MS		water	[58]
MOF-74-C		MOF-74		odorous organic contaminants		SPME		GC-MS		water	[59]
Co@ZIF-67-C		ZIF-67		PAHs		SPME		HPLC-UV		water	[60]
Co-NPC		ZIF-67		fluorouracil and phenobarbital		SBSE		HPLC-UV		human serum and urine	[62]
MPC		MOF-67		estrogen		DMSPE		GC-MS		water	[63]

MOF-C: metal-organic framework derived nanoporous carbon; ZIF-8-NC: zeolitic imidazolate framework-8 derived nitrogen-containing porous carbon; MNC: magnetic nanoporous carbon; BMZIF: bimetallic zeolitic imidazolate framework; BTC: 1,3,5-benzenetricarboxylic acid; HPC: hierarchically porous carbon; CNTCs: carbon nanotube cages; NPC: nanoporous carbon; HCNC: hollow carbon nanocube; MIL: materials of institut lavoisier; MPC: magnetic porous carbon. PAHs: polycyclic aromatic hydrocarbons; PCBs: polychlorinated biphenyls; BTEX: benzene series compounds. dSPE: dispersive solid phase extraction; MSPE: magnetic solid phase extraction; SBSE: stir bar sorptive extraction; DMSPE: dispersive micro solid phase extraction. FAAS: flame atomic absorption spectroscopy; μECD: micro-electron capture detection.

## 5 结论与展望

MOFs衍生物制备简便,具有独特的微观结构以及优异的性能,是一种极具应用前景的吸附剂,已在复杂环境样品、食品以及生物样品中目标物的分离分析方面得到了广泛应用。目前有上千种已知MOFs,而关于MOFs衍生物的研究相对较少,因此MOFs衍生物未来将有广阔的发展前景。虽然目前已开发了多种MOFs衍生物应用于样品前处理领域,但是它们对目标分析物的靶向吸附能力都较差,根据目标物的特性来精准设计或功能化MOFs衍生物,是极具挑战性和应用潜力的新方向。例如,可以通过使用SiO_2_、聚合物、表面活性剂等^[66]^设计特定孔结构的MOFs衍生物,以选择性吸附相应分子尺寸的目标分析物;或者针对目标分析物的官能团特点,在MOFs衍生物表面修饰能与其产生相互作用的官能团。此外,探索一些可以增大MOFs衍生物比表面积的方法,对前处理技术的应用也起着积极推动的作用。例如,使用KOH进行化学活化,可以显著增大MOFs衍生物的比表面积^[67]^。An等^[68]^报道了通过KOH活化后,MAF-6衍生物的比表面积可高达3123 m^2^/g,而没有KOH活化的MAF-6衍生物,其比表面积仅为1484 m^2^/g。MOFs衍生物的低产率(通常是13%~21%)也是限制其大规模应用的原因之一^[9]^。尽管通过热解MOFs和其他碳源的复合物可以提高其产率,但是额外碳源的存在会影响MOFs衍生物的微观结构和吸附活性位点,从而影响其吸附性能。因此,与MOFs相比,MOFs衍生物在前处理领域的发展和应用还有更多可探索的空间。
